# Physical Characterization of Mouse Deep Vein Thrombosis Derived Microparticles by Differential Filtration with Nanopore Filters

**DOI:** 10.3390/membranes2010001

**Published:** 2011-12-27

**Authors:** Antonio Peramo, Jose A. Diaz

**Affiliations:** 1 Department of Oral and Maxillofacial Surgery, 1150 W Medical Drive, MSRBII A560, University of Michigan, Ann Arbor, MI 48109, USA; 2 Department of Surgery, Section of Vascular Surgery, University of Michigan, Ann Arbor, MI 48109, USA; Email: josediaz@umich.edu

**Keywords:** membrane filtration, nanopore filter, thrombosis, deep vein thrombosis, microparticle

## Abstract

With the objective of making advancements in the area of pro-thrombotic microparticle characterization in cardiovascular biology, we present a novel method to separate blood circulating microparticles using a membrane-based, nanopore filtration system. In this qualitative study, electron microscopy observations of these pro-thrombotic mouse microparticles, as well as mouse platelets and leukocytes obtained using a mouse inferior vena cava ligation model of deep-vein thrombosis are presented. In particular, we present mouse microparticle morphology and microstructure using SEM and TEM indicating that they appear to be mostly spherical with diameters in the 100 to 350 nm range. The nanopore filtration technique presented is focused on the development of novel methodologies to isolate and characterize blood circulating microparticles that can be used in conjunction with other methodologies. We believe that determination of microparticle size and structure is a critical step for the development of reliable assays with clinical or research application in thrombosis and it will contribute to the field of nanomedicine in thrombosis.

## 1. Introduction

Thrombosis is an area of cardiovascular research that would substantially benefit of novel technological approaches, in particular, studies focusing on the characterization and manipulation of blood microparticles (MPs). Our focus is on venous thromboembolism (VTE) [1]. VTE include deep vein thrombosis (DVT) and pulmonary embolism (PE) and together account for approximately 900,000 new cases annually [2]. Microparticles are small vesicles that are generated (released) from a variety of cells of different origin, including leukocytes, platelets and endothelial cells. In general, it is thought that they appear as small cellular fragments, which in the past had been thought to be just cellular debris. However, it was demonstrated that the MPs were generated when cells were activated or suffered apoptosis [3]. The mechanisms on how MPs are released *in vivo* are unknown, but some studies are shown that, for example, shear stress levels may stimulate MPs release from endothelial cells [4]. The activation of leukocytes, platelets and endothelial cells promotes a general membrane content redistribution that will ultimately derive in MPs but the majority of them appear to be derived from platelets [5,6,7]. This membrane content redistribution is done by the translocation of anionic phospholipids like phosphatidylserine, from the inner to the outer membrane of the cell and is controlled by enzymes like floppase and scramblase [8]. MPs then carry membrane proteins and also microRNAs and can affect the physiology of other cells with which they interact in different ways [9,10] It is generally considered that low levels of MPs are found circulating in the blood of healthy individuals [11,12,13] and have been shown to increase in thrombotic diseases [14,15] where an intense inflammatory response occurs with the production of pro-coagulant MPs. MPs are now considered a potent pro-coagulant particle and has been suggested as possible biomarker for DVT [16,17]. 

In recent years, circulating MPs have received attention as potential biomarkers in the diagnosis and prognosis of disease, including thrombosis. However, such studies have been hampered by the lack of accurate methods for the detection and quantification of MPs. Despite the great efforts by experts in vascular biology, the field of thrombosis is facing the problem that the methods currently being used for detection and/or quantification of MPs are inadequate. They involve techniques that do not “visualize” the microparticles and, unfortunately, do not provide enough information regarding their physical characteristics, like flow cytometry [18] or functional assays [19].

In this study, MPs were generated using a well-documented mouse inferior vena cava (IVC) ligation model of DVT [20,21,22,23,24]. The justification for the use of the model is that it is considered a good model to observe increased number of microparticles and has been reported extensively elsewhere [21,22,24]. Accompanying the MPs observations we also present electron microscopy observations from mouse platelets and leukocytes, cells that participate in thrombus formation. With a focus on the development of novel methodologies to isolate and characterize MPs, we developed an innovative method to isolate and separate the particles using commercially available nanopore filters of varied porosities. The use of these type of membranes may provide an alternative procedure for the separation of the MPs from the rest of the blood cells, possibly limiting or eliminating the need of ultracentrifugation procedures and be used as intermediate or complimentary step in other techniques currently in use for their study, including flow cytometry. The technique presented also suggests the need for further development of more specialized filters and membranes for applications in nanomedicine.

It is important to note that most of these previous works have studied MPs in human samples. To our knowledge, visualization and characterization of these MPs in mice has not been previously performed. In addition, we are not aware of any study in which MPs have been visualized by TEM or SEM after artificial induction of the thrombotic event in a mouse model.

## 2. Experimental Section

### 2.1. Blood Collection from Mice and DVT Ligation Procedure

**Figure 1:**
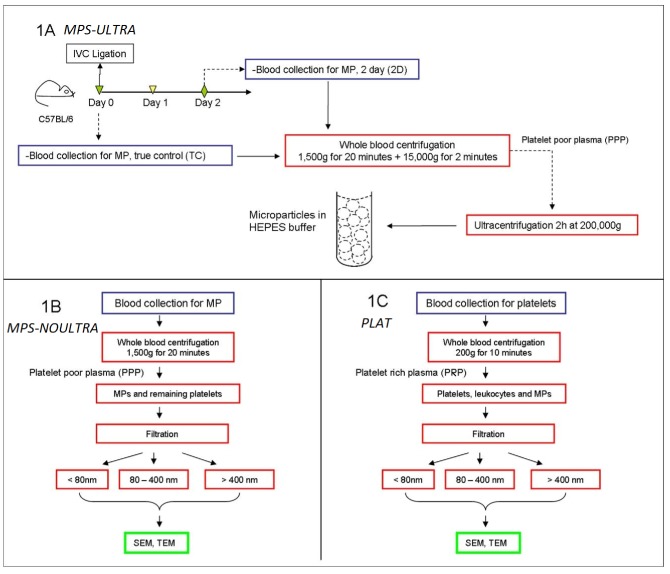
(**1A**) indicates how cells and MPs were extracted from control mice and from mice subjected to the IVC ligation procedure. IVC ligation is performed to generate deep vein thrombosis in C57BL/6 mice. Blood was drawn 2 days after surgery and MPs extracted. Simultaneously, blood was also collected from true control animals (no IVC ligation). This is the regular procedure to obtain microparticles and involves two centrifugation steps (MPS-ULTRA); (**1B** and **1C**) show a novel alternative procedure for MP separation using nanopore filters to separate platelets and MPs. This eliminates the two ultracentrifugation steps shown in (**1A**). The filtration separates the MPs into three ranges: below 80 nm, between 80 to 400 nm and above 400 nm. The pore size of the filters was 1 µm, 400 nm and 80 nm. (**1C**) shows the procedure to obtain platelets, in this case by collecting platelet rich plasma.

Twenty C57BL/6 male mice (8-10 weeks old, Charles River Laboratories, Wilmington, MA, USA) weighing 20-25 grams were used and blood was collected in acid citrate dextrose (ACD, Sigma-Aldrich, St Louis, MO, USA) to obtain blood cells and MPs from two groups: true control (TC) mice, where no IVC ligation was performed; and mice subjected to the IVC ligation procedure, from now on referred to as (2D), because the extraction of the MPs took place 2 days after ligation [21,22]. A flow chart is shown in Figure 1. All mice were housed and cared for by the University of Michigan Unit for Laboratory Animal Medicine and were free of pathogens. The University of Michigan Committee on Use and Care of Animals approved this research protocol. Common chemical reagents were purchased from Sigma-Aldrich, St Louis, MO, USA). 

### 2.2. MPs Separation Using Ultracentrifugation: (MPS-ULTRA) (Figure 1A)

This protocol is one of the regular separation methods for MPs [24]. 0.5 mL whole blood draw was extracted from each mouse by terminal cardiac puncture and transferred into 10% ACD. Platelet poor plasma (PPP) was obtained by centrifuging blood at 1,500 g and 23 °C for 20 minutes. Then, samples were centrifuged once more for 2 minutes at 15,000 g to remove contaminant cells from the plasma. The final PPP (200 µL) obtained from each mouse was then diluted 1:3 with HEPES buffer (pH 7.4). These samples were next centrifuged for 2 hours at 200,000 g and 4 °C to separate the microparticles using an XL-70 ultracentrifuge (Beckman Instruments, Palo Alto, CA, USA) for final MPs separation. The supernatant is removed, and the pellet MPs re-suspended in 300 µL HEPES buffer (pH 7.4). This suspension was then directly used for sample preparation for TEM and SEM.

### 2.3. MPs Separation Using Filters: (MPS-NOULTRA) (Figure 1B)

0.5 mL of whole blood was drawn from each mouse, by terminal cardiac puncture, with a 26 g needle into a 1 mL syringe pre-loaded with 0.05 mL of ACD [7]. We used ACD as anticoagulant because of its lower platelet activation. Platelet poor plasma (PPP) was obtained by centrifuging blood at 1,500 g and 23 °C for 20 minutes. The PPP obtained was then fixed in 2% formalin for 30 minutes. The samples were then filtered using 1 µm, 400 nm and 80 nm filters. The filters (Nuclepore Track-Etch, Whatman, Piscataway, NJ, USA) are made of polycarbonate and were used with a Millipore filter holder (Whatman, Piscataway, NJ, USA). The filters were used “as is” with no modification or treatment. Note that this protocol does not include ultracentrifugation.

### 2.4. Platelet Separation (PLAT) (Figure 1C)

0.5 mL of whole blood draw was extracted from each mouse by terminal cardiac puncture and transferred into 10% acid citrate dextrose (ACD). Platelet rich plasma (PRP) was obtained by centrifuging blood at 200 g at room temperature for 10 min. Cell suspensions were then used for TEM and SEM analysis.

### 2.5. Scanning Electron Microscopy

After re-suspension in HEPES buffer and for all separation procedures, MPs, platelets, or leukocytes were fixed for 30 minutes in 2% formalin. The suspensions with cells were each separated in three equal aliquots. One aliquot was filtered with an 80 nm filter; a second aliquot with a 400 nm filter and the third aliquot with a 1 µm filter. The same procedure was repeated for the MPs suspensions. Samples were then dehydrated through a graded series of ethanol and dried overnight with hexamethyldisilazane (Ted Pella, CA, USA). A thin film of gold was deposited on the samples using a common gold sputterer. SEM micrographs were taken with an Amray 1910 Field Emission SEM.

### 2.6. Transmission Electron Microscopy

MPs separated using MPS-ULTRA procedure and platelets or leukocytes separated using PLAT procedure were pelleted and then fixed with 2.5 percent glutaraldehyde in 0.1 M Sorensen’s buffer (Electron Microscopy Science, Hatfield, PA, USA), pH 7.4. The fixed cell pellets were then encapsulated in Histogel (Thermo Scientific). After several buffer rinses, they were post-fixed for one hour in one percent osmium tetroxide (Electron Microscopy Science Hatfield, PA, USA) in the same buffer. Next, they were rinsed in Sorensen’s buffer and then dehydrated in ascending concentrations of ethanol. They were then treated with propylene oxide (Electron Microscopy Science Hatfield, PA), and infiltrated and embedded in Epon epoxy resin (Polysciences Inc., Warrington, PA, USA). Semi-thin sections were stained with toluidine blue for tissue orientation. Selected areas were ultra-thin sectioned 70 nm in thickness, applied to copper grids and post-stained with uranyl acetate and lead citrate (Electron Microscopy Science, Hatfield, PA, USA). They were examined using a Philips CM100 electron microscope at 60 kV. Images were recorded digitally using a Hamamatsu ORCA-HR camera system operated using AMT software (Advanced Microscopy Techniques Corp., Danvers, MA, USA).

## 3. Results

### 3.1. Mouse MP Sizes in Non-DVT

**Figure 2:**
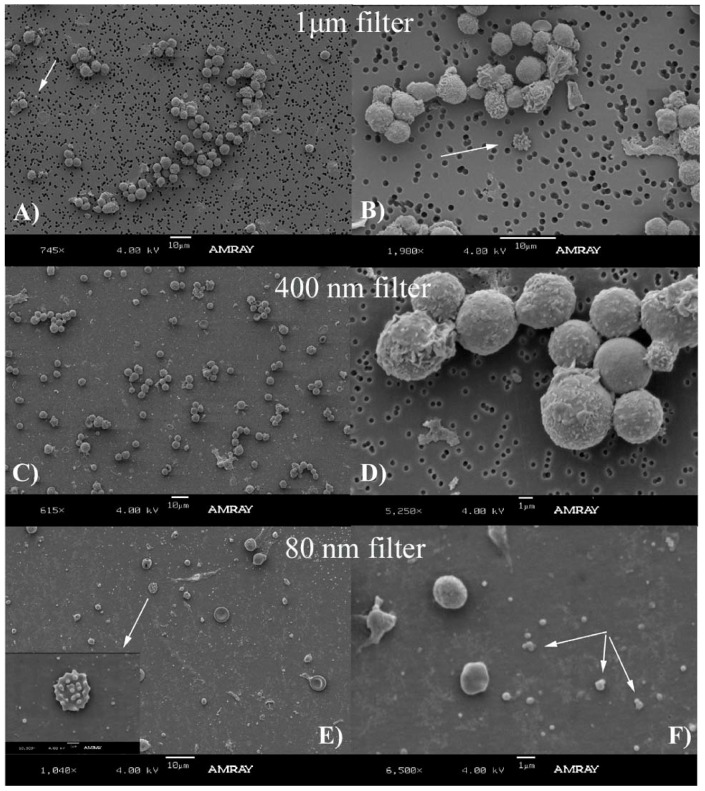
This figure shows leukocytes and accompanying MPs obtained using the platelet separation(PLAT) protocol (described in detail in Figure 1C). The suspensions of platelet rich plasma were filtered and separately deposited in 1 µm (**A** and magnification **B**), 400 nm (**C** and magnification in **D**) and 80 nm filters (**E** and magnification in **F**). The cells and MPs were extracted from true control samples where no DVT ligation procedure was performed. Indicated with white arrows are the particles deposited in the filters, some of them aggregated. Note that there are no isolated (without aggregation) MPs left in the big pore filters (B, D) but non-aggregated particles are visible in the 80 nm filter (F).

**Figure 3:**
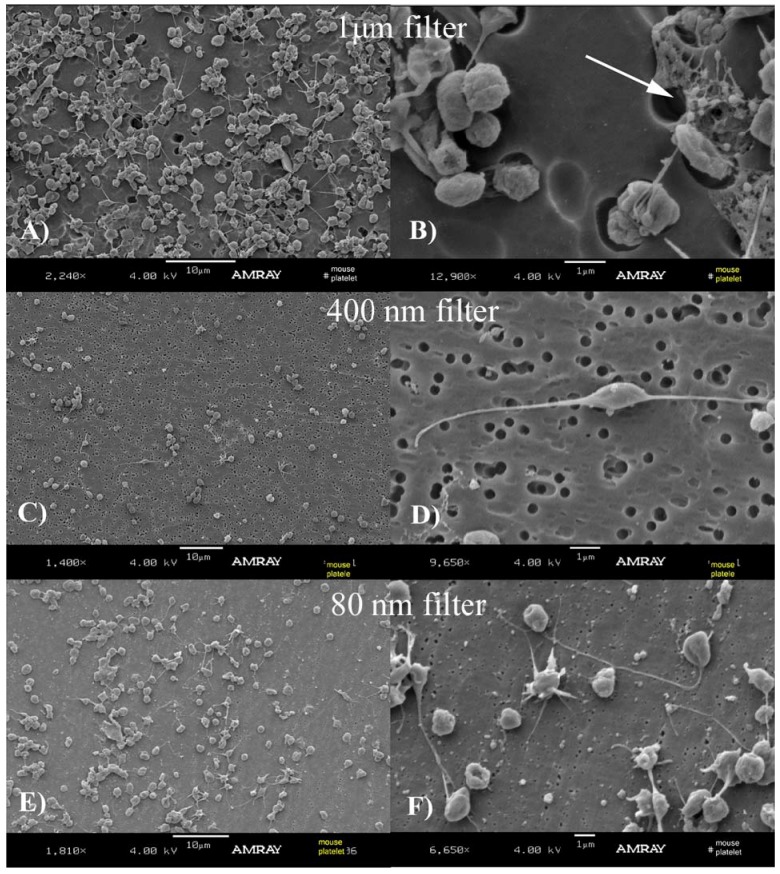
Platelets and MPs obtained using platelet separation (PLAT) protocol (described in detail in Figure 1C). The suspensions of platelet rich plasma were separately filtered through 1 µm (**A**), 400 nm (**C**) and 80 nm filters (**E**) (magnification of the same shown respectively in **B**, **D** and **F**). As indicated in Figure 2, this demonstrates that most MPs have been eluted using the 1 µm and 400 nm filters, but have been retained by the 80 nm. The cells and MPs were extracted from true controls where no ligation procedure was performed.

Following the PLAT protocol, our studies using separation by filters and electron microscopy revealed that mouse MP sizes from TC are under 1 µm in diameter and that their shape is spherical or near spherical. Our results were separated in Figure 2 (MPs and leukocytes) and Figure 3 (MPs and Platelets). The SEM images obtained from a 1 µm filter demonstrated the presence of leukocytes and a few particles were observed that seem to be aggregated (Figure 2A,B) and the presence of platelets and a few aggregated MPs were observed (Figure 3A,B). 

The SEM images obtained from a 400 nm filter (Figure 2C,D and Figure 3C,D) and 80 nm filter (Figure 2E,F and Figure 3E,F) showed the most MPs. The lack of MPs using 1 µm filters indicate that MPs are washed through the pores because a majority of them have smaller diameters. This is confirmed in Figure 2C, where several particles are observed. Figure 2D is a close-up image of the leukocytes. The structure with several MPs observed in image B is also seen in image E. Aggregated MPs were also found (image F).

### 3.2. Mouse MPs in DVT

**Figure 4:**
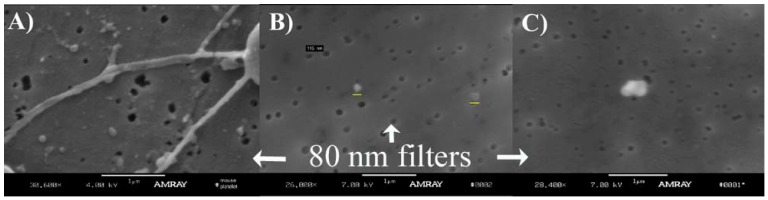
Shown are MPs in the areas close to platelets from true controls (TC) mice (**A**) on a 80 nm filter (PLAT procedure, described in Image 1C); and TC (**B**) and day 2 post ligation (2D) mice (**C**) samples (using the MPS-ULTRA procedure, described in Image 1A). The low concentration of MPs obtained during separation and centrifugation (**B** and **C**) prevented any definitive conclusion regarding differences in morphology of the MPs from true controls (**A** and **B**) versus 2D (**C**).

An attempt to compare the aspect of the MPs between TC and 2D was made (Figure 4). No conclusive differences were observed between MPs in (**A**) (TC platelet extracted using PLAT procedure, image 1); (**B**) (TC MPs extracted using MPS-ULTRA); and (**C**) (2D MPs extracted using MPS-ULTRA). The low concentration of MPs obtained for samples (**B**) and (**C**) prevented to reach any definitive conclusion regarding differences in morphology of the TC MPs and the 2D MPs.

**Figure 5:**
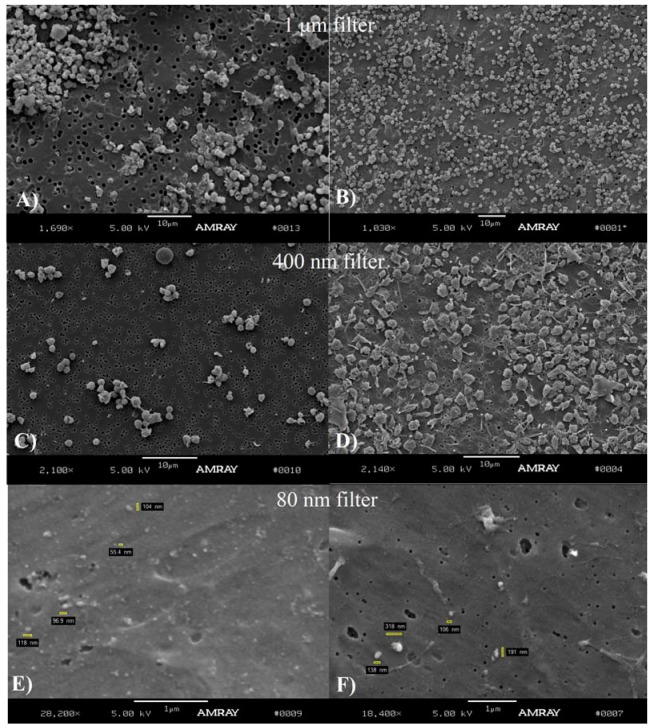
Overall observation of MPs obtained from TC and 2D post-ligation samples without ultracentrifugation (MPS-NOULTRA, as described in Figure 1B). (**A** and **B**) MPs and platelets deposited on a 1 μm filter. The eluents of the same suspensions were filtered using a 400 nm filter (**C** for TC, **D** for 2D) and an 80 nm filter **E** (TC) and **F** (2D). While a quantitative estimation cannot be done, the results suggest that the filtration procedures preserve a higher number of MPs, compared with the regular ultracentrifugation procedures shown in Figure 4. The size of the particles retained in the filters are in the range of 100 to 300 nm.

We observed low concentration of MPS on the filters. To increase this concentration and to facilitate imaging, a procedure without centrifugation was devised (MPS-NOULTRA, Figure 5). In this particular case, samples were obtained from blood at 2D post-ligation. MPs were obtained from the eluent of the filtration with a 1 µm filter resting on top of a 400 nm filter and MPs were obtained in the eluent of the filtration with the 400 nm filter resting on top of an 80 nm filter, indicating that MPs were deposited on the filters with increased concentrations.

**Figure 6:**
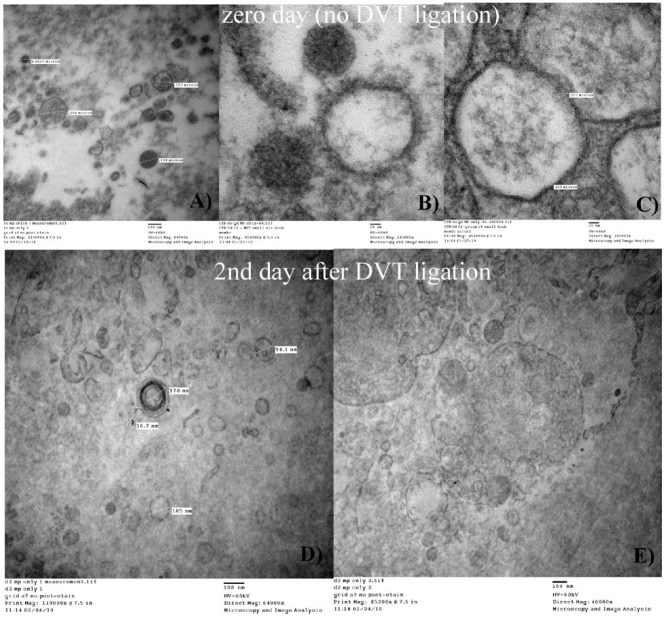
Composite TEM micrographs of MPs from TC mice (**A**, **B** and **C**) and mice 2 days post-ligation (**D** and **E**) using the MPS-ULTRA procedure (described in Figure 1A). Note the regular circular morphology of the vesicles, the diameters below 400 nm and the observation that some seem to be shells and others solid bodies (this difference is more noticeable in A and B).

Some micrographs show distorted vesicles, in the form of revolution ellipsoids, probably due to the SEM preparation. Most of the vesicles present in the leukocytes and platelets micrographs had sizes in the range of 100 to a little over 400 nm. This mostly spherical geometry of the MPs was also confirmed with the TEM micrographs in Figure 6. 

Using TEM micrographs of TC and 2D MPs extracted by MPS-ULTRA procedure, we found that MPs appear to be very dark or gray and some contain a small amount of internal material, but most of them seem to be empty (Figure 6). Discounting the possible darkening effect of the staining and the precise orientation where the samples were sectioned, is clear that some MPs have a compact, “hard” interior while others can primarily be characterized as “shells”. Most of these MPs (TC or 2D) look spherical and their diameters are in the range of 100 to 250 nm.

## 4. Discussion

In general, circulating MPs are considered small vesicles (less than 1 micrometer) consisting of a small amount of cytoplasm and a very well structured plasma membrane characterized by a controlled transverse lipid distribution termed “rafts” [5]. Given the different origins of the MPs as well as their increased presence during thrombosis, there is a need to perform additional analysis of the structure and physical characteristics of these vesicles. Despite the multiple efforts there is not a universal method that is able to provide all of the information needed to thoroughly study MPs.

Methods typically used for determination of size do not provide visual evidence of the characteristics of the particles and there are increased discussions regarding what MPs size and number these techniques actually provide [25]. These issues are compounded in the case of animal models, where the information available is more limited. In addition, during the historical period where initial SEM and TEM observations of mouse platelets and leukocytes were performed (around 30 years ago), MPs were not studied in detail [26].

We note that we used standard protocols for extraction of microparticles published in the literature and commonly used by one of the authors, including the type of fixatives, with the innovative modification of using filters for their separation by size. We initiated our analysis of MPs using an animal model of DVT where thrombosis can be surgically induced in mice, which permits the analysis of the generated MPs during thrombus formation, in this case at day two. This controlled analysis cannot be performed in humans. We are not aware of previous publications where pro-thrombotic microparticles from mice were studied using SEM and TEM. The selection of studying blood from an animal at two days after IVC ligation is based on the fact that MP concentrations are known to be the highest at this particular time point, based on previous studies [24]. From day 2 on MP counts steadily decrease, although it is still possible to obtain and separate MPs at 14 days post-surgery. 

Previous investigations have suggested that MPs are very heterogenic in size [27]. MPs largest size in humans is considered to be about 1.5 µm, while the mean size of platelets is 1.3 µm. However, in this study, we were essentially unable to find mouse MPs over 500 nm. While our study was conducted with mouse MPs, this is broad agreement with current studies in humans, indicating that they are smaller [28]. 

On the other hand, small MPs may overlap in size with a group of particles known as exosomes (40-80 nm), which are derived from multivesicular bodies and have different biological origin and function [29]. We did not attempt to analyze MPs with sizes under 80 nm because of substantial limitations in SEM imaging capabilities, but we suggest that our methodology, with appropriate nanopore filters, can perhaps be used for exosome separation. It is important to note that in our experimental procedures we did not separate the extracted MPs by their cellular origin or by their membrane antigen expression and hence is always possible that some other particles can be present in the images presented. However, the homogenous aspect, size and similarity of the particle population obtained and the strict use of published protocols for the preparation and extraction of the MPS indicates that the methodology is valid. For this reason, our results provide an average, broad view of the characteristics of the non-manipulated MPs. In other words, regardless of the origin, mouse MPs appear to be within the 100 to 350 nm diameter range. 

Our experiments were always performed with fresh samples but it has been reported that cold storage and ultracentrifugation may differentially affect MPs of different biological origins [30]. Is not clear whether they also affect MPs size and morphology. Our observations, presented in Figure 2, Figure 3, Figure 4, Figure 5, Figure 6, indicate that mouse MPs size is smaller than what has previously been reported in the literature. We can hypothesize that mouse MPs are smaller based just on the fact that this is another species, or the fact that the information in the literature may have been overestimated mostly because of the non-visual techniques used. We did not attempt to make a quantitative analysis of the relative number of MPs found on each filtration process, but qualitatively it seemed that there were MPs that did not elute from the 400 nm filters, indicating that some MPs may lie in the 400 nm or bigger diameter range. 

We also observed that the concentration of MPs visible in the filters during some of the filtration procedures were low. In addition, all platelets were retained in the 1 µm filter thus we concluded it could be possible to get increased concentrations of MPs by using a filtration, instead of an ultracentrifugation, method (MPS-NOULTRA). To verify these assessments, an experiment was performed using MPs obtained from blood at 2D post-ligation without ultracentrifugation (MPS-NOULTRA), obtained from a poor plasma platelet suspension and separated using a sequential filtration process. As mentioned, the observations confirm that the size of the MPs is mainly in the 100 to 350 nm range. In addition, the technique of sequential filtering appears to produce higher concentrations of MPs than when ultracentrifugation is used, suggesting that this technique (results shown in Figure 5), used with more appropriate filters in clinical settings for quantification of MPs in humans may provide a more accurate method to separate MPs than the current ultracentrifugation technique. It should be noted that there are currently no “standard” techniques for MPs separation used by all laboratories involved in thrombosis research. This has been mentioned before, as “there is no inter-laboratory agreement on the pre-analytic preparation of plasma samples” [30] and has been compared by Jy *et al.* [31], and this is another reason why we conducted this study.

Another plausible explanation for the apparent MP size overestimation found in the literature is the possible aggregation of MPs with themselves or with cells, as shown in Figure 2. This aggregation can be due to the SEM preparation process, but is also an indication that these vesicles could form these groups in suspension and perhaps favoring or inducing formation of the thrombi. This aggregation may be due to the overall surface charge density of the MPs and the composition of the buffers used during re-suspension. As mentioned before, MPs are described as carrying a cytoskeleton surrounded by a membrane consisting of a phospholipid bilayer, presenting a higher density of negatively charged phospholipids than regular cells, particularly phosphatidylserine, on its outer membrane layer. When blood cells are activated, the distribution of phospholipids is disturbed resulting in the externalization on the outer leaflet of the membrane amino phospholipids which are more negative at a physiologic pH. This would suggest that, if aggregation occurs, it can possibly be reduced by using a slightly below neutral pH buffer during MPs extraction and separation.

The work presented is qualitative and no attempt was made to obtain quantitative data or statistical data due to our limited number of samples. In general our findings suggest that mouse circulating MPs have a spherical or near spherical shape and sizes in the 100 to 350 nm range. When using filters to select particles over 1 µm we were not able to observe any, although this may be due to a low ratio of MPs of this size respect to MPs of smaller sizes. As in our case, spherical or near spherical MPs have been observed previously and recent analysis using atomic force microscopy also appears to confirm these characteristics [32]. The average size observed in our experiments appears in agreement with the observation that MPs may be derived from internal bodies of the platelets or from lipid rafts [33] as recent studies focusing on the detection and isolation methods of vesicles are also reporting [34,35,36]. 

TEM micrographs of MPs indicated that their size is mostly in the previously mentioned range. Due to our limited number of samples we were unable to discriminate whether TC MPs and 2D MPs have a significantly different size and they also do not appear to be morphologically different. However, it is clear that with both regular cell extraction procedure (PLAT) and the regular MP extraction (MPS-ULTRA) procedure, three types of MPs were observed, as seen in Figure 6, some solid (dark); some empty and others with a small amount of material. At this stage, however, no attempt was made to separate the MPs by its origin and the pictures show an average view of the MPs and possibly released cell granules.

## 5. Conclusions

In conclusion, the data presented will help advance the exploration into MPs size and provides visual evidence of their morphology. We believe the technical approach of using filters to separate MPs should be explored in more detail, particularly if used in combination with other novel technologies for the study of these biological particles [31]. We limited our study to commercially available filters of specific ranges, but membrane developments targeting this type of particles could be of interest.

## References

[1] Heit J.A. (2005). Venous thromboembolism: Disease burden, outcomes and risk factors. J. Thromb. Haemost..

[2] Diaz J., Wakefield T., Wittens C. (2009). Intrinsic and Extrinsic Causes of Thrombosis in the Superficial and Deep Venous System in Innovative Treatment of Venous Disease.

[3] Burnier L., Fontana P., Kwak B.R., Angelillo-Scherrer A. (2009). Cell-derived microparticles in haemostasis and vascularmedicine. Thromb. Haemost..

[4] Boulanger C.M., Amabile N., Guerin A.P., Pannier B., Leroyer A.S., Mallat C.N., Tedgui A., London G.M. (2007). *In vivo* shear stress determines circulating levels of endothelial microparticles in end-stage renal disease. Hypertension.

[5] Ahn E.R., Lander G., Jy W., Bidot C.J., Jimenez J.J., Horstman L.L., Ahn Y.S. (2004). Differences of soluble CD40L in sera and plasma: Implications on CD40L assay as a marker of thrombotic risk. Thromb. Res..

[6] Wakefield T.W., Myers D.D., Henke P.K. (2008). Mechanisms of venous thrombosis and resolution. Arterioscler. Thromb. Vasc. Biol..

[7] Zwaal R.F.A., Schroit A.J. (1997). Pathophysiologic implications of membrane phospholipid asymmetry in blood cells. Blood.

[8] Zwaal R.F., Comfurius P., Bevers E.M.  (2005). Surface exposure of phosphatidylserine in pathological cells. Cell. Mol. Life Sci..

[9] Deregibus M.C., Cantaluppi V., Calogero R., Lo Iacono M., Tetta C., Biancone L., Bruno S., Bussolati B., Camussi G. (2007). Endothelial progenitor cell derived microvesicles activate an angiogenic program in endothelial cells by a horizontal transfer of mRNA. Blood.

[10] Yuan A., Farber E.L., Rapoport A.L., Tejada D., Deniskin R., Akhmedov N.B., Farber D.B. (2009). Transfer of microRNAs by embryonic stem cell microvesicles. PLoS One.

[11] Piccin A., Murphy W.G., Smith O.P. (2007). Circulating microparticles: Pathophysiology and clinical implications. Blood Rev..

[12] Combes V., Simon A.C., Grau G.E., Arnoux D., Camoin L., Sabatier F., Mutin M., Sanmarco M., Sampol J., Dignat-George F. (1999). *In vitro* generation of endothelial microparticles and possible pro-thrombotic activity in patients with lupus anticoagulant. J. Clin. Invest..

[13] Berckmans R.J., Neiuwland R., Boing A.N., Romijn F.P., Hack C.E., Sturk A. (2001). Cell-derived microparticles circulate in healthy humans and support low grade thrombin generation. Thromb. Haemost..

[14] Rubin O., Crettaz D., Tissot J.D., Lion N. (2010). Microparticles in stored red blood cells: Submicron clotting bombs?. Blood Transfus..

[15] Chironi G.N., Boulanger C.M., Simon A., Dignat-George F., Freyssinet J.M., Tedgui A. (2009). Endothelial microparticles in diseases. Cell Tissue Res..

[16] Rectenwald J.E., Myers D.D., Hawley A.E., Longo C., Henke P.K., Guire K.E., Schmaier A.H., Wakefield T.W. (2005). D-dimer, P-selectin, and microparticles: Novel markers to predict deep venous thrombos. A pilot study. Thromb. Haemost..

[17] Simak J., Gelderman M.P. (2006). Cell membrane microparticles in blood and blood products: Potentially pathogenic agents and diagnostic markers. Transfus. Med. Rev..

[18] Abrams C.S., Ellison N., Budzynski A.Z., Shattil S.J. (1990). Direct detection of activated platelets and platelet-derived microparticles in humans. Blood.

[19] Ho W.K. (2010). Deep vein thrombosis-risks and diagnosis. Aust. Fam. Physician.

[20] Day S.M., Reeve J.L., Myers D.D., Fay W.P. (2004). Murine thrombosis models. Thromb. Haemost..

[21] Bouzeghrane F., Zhang X., Gevry G., Raymond J. (2008). Deep vein thrombosis resolution is impaired in diet-induced type 2 diabetic mice. J. Vasc. Surg..

[22] Nosaka M., Ishida Y., Kimura A., Kondo T. (2010). Immunohistochemical detection of MMP-2 and MMP-9 in a stasis-induced deep vein thrombosis model and its application to thrombus age estimation. Int. J. Legal Med..

[23] Wojcik B.M., Wrobleski S.K., Hawley A.E., Wakefield T.W., Myers D.D., Diaz J.A. (2011). Interleukin-6: A potential target for post-thrombotic syndrome. Ann. Vasc. Surg..

[24] Ramacciotti E., Hawley A.E., Farris D.M., Ballard N.E., Wrobleski S.K., Myers D.D., Henke P.K., Wakefield T.W. (2009). Leukocyte- and platelet-derived microparticles correlate with thrombus weight and tissue factor activity in an experimental mouse model of venous thrombosis. Thromb. Haemost..

[25] Freyssinet J.-M., Toti F. (2010). Membrane microparticle determination: At last seeing what’s being sized!. J. Thromb. Haemost..

[26] Orenstein J.M., Shelton E. (1976). Surface topography of leukocytes* in situ*: Cells of mouse peritoneal milky spots. Exp. Mol. Pathol..

[27] Perez-Pujol S., Marker P.H., Key N.S. (2007). Platelet microparticles are heterogeneous and highly dependent on the activation mechanism: Studies using a new digital flow cytometer. Cytometry A.

[28] Lawrie A.S., Albanyan A., Cardigan R.A., Mackie I.J., Harrison P. (2009). Microparticle sizing by dynamic light scattering in fresh-frozen plasma. Vox Sanguinis.

[29] Théry C., Zitvogel L., Amigorena S. (2002). Exosomes: Composition, biogenesis and function. Nat. Rev. Immunol..

[30] Ierssel S.H., van Craenenbroeck E.M., Conraads V.M., Van Tendeloo V.F., Vrints C.J., Jorens P.G., Hoymans V.Y. (2010). Flow cytometric detection of endothelial microparticles (EMP): Effects of centrifugation and storage alter with the phenotype studied. Thromb. Res..

[31] Jy W., Horstman L.L., Jimenez J.J., Ahn Y.S., Biro E., Nieuwland R., Sturk A., Dignat-George F., Sabatier F., Camoin-Jau L. (2004). Measuring circulating cell-derived microparticles. J. Thromb. Haemost..

[32] Yuana Y., Oosterkamp T.H., Bahatyrova S., Ashcroft B., Garcia Rodriguez P., Bertina R.M., Osanto S. (2010). Atomic force microscopy: A novel approach to the detection of nanosized blood microparticles. J. Thromb. Haemost..

[33] Bodin S., Tronchere H., Payrastre B. (2003). Lipid rafts are critical membrane domains in blood platelet activation processes. Biochim. Biophys. Acta.

[34] Kang D., Oh S., Ahn S.M., Lee B.H., Moon M.H. (2008). Proteomic analysis of exosomes from human neural stem cells by flow field-flow fractionation and nanoflow liquid chromatography-tandem mass spectrometry. J. Proteome Res..

[35] van der Pol E., Hoekstra A.G., Sturk A., Otto C., van Leeuwen T.G., Nieuwland R. (2010). Optical and non-optical methods for detection and characterization of microparticles and exosomes. J. Thromb. Haemost..

[36] Zwicker J.I., Liebman H.A., Neuberg D., Lacroix R., Bauer K.A., Furie B.C., Furie B. (2009). Tumor-derived tissue factor-bearing microparticles are associated with venous thromboembolic events in malignancy. Clin. Cancer Res..

